# Does thermodynamic stability of peritoneal collagen change during laparoscopic cholecystectomies? A differential scanning calorimetry (DSC) study

**DOI:** 10.1007/s00464-014-3513-y

**Published:** 2014-04-02

**Authors:** Kamil Torres, Hanna Trębacz, Magdalena Bącik-Donica, Agnieszka Atras, Anna Torres, Zbigniew Plewa

**Affiliations:** 1 Human Anatomy Department, Laboratory of Medical Simulation, Medical University of Lublin, Jaczewskiego 4, 20-094 Lublin, Poland; 2 Department of General Surgery, District Specialist Hospital, Lublin, Poland; 3 Department of Biophysics, Medical University of Lublin, Lublin, Poland; 4 Laboratory of Biostructure, Human Anatomy Department, Medical University of Lublin, Lublin, Poland; 5 Department of General Surgery, Clinical Military Hospital No.1, Lublin, Poland

**Keywords:** Laparoscopy, Peritoneum, Calorimetry, Surgery

## Abstract

**Background:**

Carbon dioxide pneumoperitoneum used during laparoscopic surgeries alters the integrity of the peritoneum and results in denudation of the basal lamina that might cause altered immune response, inhibited fibrinolysis, hypoxia, and acidosis. The changes in the structure of pneumoperitoneum were described as bulging of mesothelial cells, irregular cell junction’s cell membrane degradation, and mesodermal edema. As denaturation of peritoneal proteins reflects overall condition of its structure and interactions with the surrounding molecules, the physical status of collagen was assessed on the basis of parameters of thermal denaturation measured by DSC method.

**Methods:**

Twenty-four female patients operated on due to cholelithiasis were enrolled in this study. Laparoscopic cholecystectomy was performed using standard four-trocar technique, and standard values of insufflated carbon dioxide pneumoperitoneum were used. After trocar placement, the first collection of peritoneal sample (sample A) was performed. The second peritoneal sample (sample B) was collected after the removal of gall bladder. Differential scanning calorimetry (Q200 calorimeter, TA Instruments) was performed on samples defrosted at room temperature.

**Results:**

In all samples of peritoneum, a nonreversible endothermal process recognized as denaturation was observed. Sample B obtained at the end of surgery did not differ from sample A obtained at the beginning in terms of all parameters under study. Temperature of denaturation in A and B was correlated only marginally, but enthalpy and specific heat were significantly correlated. The analysis of data from DSC measurements did not reveal differences in physical stability of collagen in peritoneal samples obtained at the beginning and at the end of surgery. Significant negative correlations between duration of CO_2_ pneumoperitoneum and enthalpy of denaturation in sample B were found.

**Conclusions:**

Differences in enthalpy of denaturation may reflect a quantitative relation between amount of native collagen molecules in the sample and other, non-collagenous components or impaired collagen.

Peritoneal tissue consists of collagen basal network filled with mesothelial cells that are covered by flowing thin filmy hydrating layer [1]. The influence of carbon dioxide used for pneumoperitoneum during laparoscopic surgeries on peritoneal tissue remains unclear [2, 3]. Carbon dioxide pneumoperitoneum used during laparoscopic surgeries might alter the integrity of the peritoneum that is one of the main players in the response to surgical trauma. Both chemical and physical factors connected with carbon dioxide pneumoperitoneum influence the integrity of peritoneal tissue and result in denudation of the basal lamina that might cause altered immune response, inhibited fibrinolysis, hypoxia, and acidosis [4, 5]. It has been also demonstrated that duration of CO_2_ pneumoperitoneum and insufflation pressure influences the propensity to adhesion formation [6]. Postoperative peritoneal adhesions are still one of the common surgical complications that might manifest even many years after the surgery [7]. The adhesions can cause abdominopelvic pain, small bowel obstruction, and infertility, and remain an important socio-economic problem. The direct medical cost of adhesion-related problems is comparable with surgical expenditure for rectal cancer [8].

Various studies described the changes of peritoneal tissue resulting from the use of carbon dioxide pneumoperitoneum [1, 4]. The changes in the structure of pneumoperitoneum were observed by Tarhan et al. even 43 min after the beginning of carbon dioxide insufflation. Authors described bulging of mesothelial cells, irregular cell junction’s cell membrane degradation and mesodermal edema [9]. These findings were in accordance with other studies that evaluated the influence of pneumoperitoneum on peritoneal tissue using scanning electron microscopy [10, 11]. However, we were unable to find studies that evaluated the changes in the collagenous structure of basal network. The nudity of basal lamina caused by pneumoperitoneum was described as a factor facilitating the formation of adhesions and occurrence of metastases [4]. Taking above data under consideration, we decided to evaluate the influence of carbon dioxide pneumoperitoneum used during relatively short laparoscopic surgeries on collagen molecules forming the physical basis of peritoneal tissue. As denaturation of a protein reflects overall condition of its structure and interactions with the surrounding molecules [12], the physical status of collagen was assessed on the basis of parameters of thermal denaturation measured by differential scanning calorimetry (DSC) method. It was shown previously that thermodynamic parameters obtained from DSC experiments are very sensitive to structure of collagen molecules, and any change in the conformation affects the position and shape of transition in DSC scans [13, 14]. So, studies of denaturation of collagen in peritoneum might help to bring additional data to the pathogenesis of formation of abdominal adhesions.

The purpose of the study was to assess if carbon dioxide pneumoperitoneum used during laparoscopic cholecystectomies alters the thermodynamic stability of peritoneal tissue.

## Materials and methods

Twenty-four female patients, operated on due to cholelithiasis at the General and Oncological Surgery Department of the District Specialist Hospital in Lublin, Poland, were enrolled in this study. All patients included in the study were informed about the aims of the research, and a written informed consent was obtained from each patient. The Ethical Committee at the Medical University of Lublin approved the study.

In each case cholelithiasis was confirmed by abdominal ultrasound. At the moment of surgery, no patient had symptoms of acute cholecystitis. Patients with surgical history, diabetes, and connective tissue disorders were excluded from the study.

Laparoscopic cholecystectomy was performed using standard four-trocar technique with the optic port at the umbilicus. Standard values of insufflated carbon dioxide pneumoperitoneum (12–14 mmHg) were used. After trocar placement, the first collection of peritoneal sample (sample A) was performed (1 cm^2^). Anatomical recognition of the structures within the Calot’s triangle was performed in each case. The dissection of the cystic duct and cystic artery with branches was performed. The duct and artery were clipped and cut. The gallbladder was removed from the bed using electrocoagulation from neck to fundus. The second peritoneal sample (sample B) was collected after the removal of gallbladder. The duration of pneumoperitoneum was between 29 and 65 min (mean 42 min). No malignancies were reported in pathological evaluation.

Samples of peritoneum collected during the surgery using laparoscopic scissors (non-thermal) were immersed in 0.9 % NaCl and frozen to −16 °C. Differential scanning calorimetry (Q200 calorimeter, TA Instruments) was performed on samples defrosted at room temperature. Hermetic aluminum pans with 5–7 mg of peritoneal tissue were filled with 15–20 μl of saline and temperature scanned from 35 to 85 °C at heating rate of 5 °C/min. A pan filled with 0.9 % NaCl was used as a reference. Peak temperature of transition (*T*_m_) referring to the temperature of protein denaturation, energy absorbed by the sample during transition referring to enthalpy (Δ*H*) of the process and change of specific heat of the sample (Δ*c*_p_) between the native and denaturated state were determined from thermograms using a software integrated with the calorimeter. Values of Δ*H* and Δ*c*_p_ were corrected for gram of dry mass of the sample.

Statistical analysis was performed using Statistica v. 10 (StatSoft). Differences between the samples A and B were accessed using paired *t* test. Correlations between samples A and B from the same patient were calculated using Pearson test, as well as correlations between parameters of denaturation and duration of pneumoperitoneum. The 5 % level of significance (*p* value < 0.05) was applied in all tests.

## Results

In all peritoneal samples, a nonreversible endothermal process recognized as denaturation was observed. Denaturation parameters for samples A and B are shown in Table 1. Sample B obtained at the end did not differ significantly from sample A obtained at the beginning of surgery in terms of all parameters under study.

**Table 1:** Parameters of thermal denaturation of peritoneal samples collected at the beginning (sample A) and at the end (sample B) of laparoscopic cholecystectomy

	Sample A		Sample B	
	Range	Mean (SD)	Range	Mean (SD)
*T*_m_ (^o^C)	66.5–69.1	67.9 (0.63)	66.5–69.4	67.8 (0.69)
Δ*H* (J/g)	9.9–29.1	18.9 (5.5)	4.0–32.9	18.8 (8.6)
Δ*c*_p_ (J/^o^C g)	0.32–2.55	1.36 (0.60)	0.41–3.08	1.45 (0.72)

To check if the broad range of values obtained can be attributed to individual differences between patients, correlations between samples A and B obtained from the same patient were calculated (Table 2). Temperature of denaturation in A and B correlated only marginally, but enthalpy and specific heat correlated significantly, so one can assume that the scatter of data reflects individual differences between patients rather than a stochastic nature of the measured process.

**Table 2:** Correlations between samples A and B from the same patient

	*r*	*p* value
*T*_m_ (^o^C)	0.4349	0.060
Δ*H* (J/g)	0.6521	0.003
Δ*c*_p_ (J/^o^C g)	0.4771	0.033

The analysis of data from DSC measurements did not reveal differences in physical stability of collagen in peritoneal samples obtained at the beginning and at the end of surgery. However, when duration of surgery was analyzed, significant negative correlations between duration of CO_2_ pneumoperitoneum and enthalpy of denaturation in samples B were found (Table 3). There was also a marginal positive correlation between duration of pneumoperitoneum and temperature of denaturation.

**Table 3:** Pearson correlation between duration of pneumoperitoneum (*t*) and parameters of denaturation (*x*) in sample B obtained at the end of surgery

	Regression equation	*r*	*p* value
*T*_m_ (^o^C)	*x* = 66.45 + 0.0321*t*	0.4887	0.0547
Δ*H* (J/g)	*x* = 38.53 − 0.503*t*	−0.6353	0.0082
Δ*c*_p_ (J/^o^C g)	*x* = 2.377 − 0.020*t*	0.2960	0.2960

## Discussion

The thermal process analyzed in the study is attributed to denaturation of collagen, the main structural protein of the peritoneal tissue. Similar values of denaturation temperatures are reported in our previous work for peritoneal samples [15] and for samples from other collagen-based tissues [16–18]. The process of thermal activation of collagen involves rupture of hydrogen bonds coupling the three α-chains and a rearrangement of the triple helix into a random chain configuration [12, 13, 16], and differences in thermodynamic parameters of denaturation for proteins are believed to reflect the differences in their native states [12, 14, 16]. Parameters of thermal transition of collagen in tissues were shown to be sensitive to the level of hydration of collagen molecules, cross-links within and between molecules and to amount and character of side-chains exposed to the surrounding medium during unfolding [12–14, 16]. Moreover, differences in enthalpy of denaturation may also reflect a quantitative relation between amount of native collagen molecules in the sample and other, non-collagenous components or impaired collagen.

The evaluation of calorimetric parameters presented in our study did not reveal any changes between the samples collected at the beginning and end of pneumoperitoneum. On the other hand, enthalpy of collagen denaturation in samples collected at the end of surgery tend to decrease along with increasing duration of operation and prolonged exposure of the peritoneum to change physical and chemical environment. It is presumable that this enthalpy decrease results from increasing fraction of impaired collagen molecules in the sample.

The changes in the basal lamina and connective tissue evaluated using electron microscopic scanning described by other authors occurred 30 min and more from the beginning of insufflation [9]. The study performed by Liu identified changes in the mesothelial cells just after the filling of peritoneal cavity with CO_2_, presence of intercellular spaces after 30 min and denudation of the basement membrane after 60 min using transmission electron microscopy and scanning electron microscopy [1]. This implicates that the denudation of basal lamina occurred after 30 min of pneumoperitoneum or later and the real time of CO_2_ exposure on the basal lamina might not take place in our study. The structural changes in the peritoneum resulting from mechanic-physical activity of carbon dioxide pneumoperitoneum have been reported to increase the peritoneal adhesion potential and therefore be the key point in intraperitoneal adhesion formation [5].

However, we plan to extend our study on a group of patients where the time of carbon dioxide pneumoperitoneum exposure will be longer. We expect that DSC measurements of thermodynamics of collagen denaturation would put a new insight into the impact of laparoscopic procedures on the pathogenesis of formation of abdominal adhesions.
